# Childbirth Induced Posttraumatic Stress Syndrome: A Systematic Review of Prevalence and Risk Factors

**DOI:** 10.3389/fpsyg.2017.00560

**Published:** 2017-04-11

**Authors:** Sharon Dekel, Caren Stuebe, Gabriella Dishy

**Affiliations:** ^1^Department of Psychiatry, Harvard Medical SchoolBoston, MA, USA; ^2^Department of Psychiatry, Massachusetts General HospitalCharlestown, MA, USA

**Keywords:** postpartum PTSD, childbirth, systematic review, posttraumatic stress, delivery, obstetrics, psychopathology, resilience

## Abstract

**Background:** Posttraumatic stress related with the childbirth experience of full-term delivery with health outcomes has been recently documented in a growing body of studies. The magnitude of this condition and the factors that might put a woman at risk for developing childbirth-related postpartum posttraumatic stress disorder (PP-PTSD) symptoms are not fully understood.

**Methods:** In this systematic review of 36 articles representing quantitative studies of primarily community samples, we set to examine PP-PTSD prevalence rates and associated predictors with a focus on the role of prior PTSD and time since childbirth.

**Results:** A significant minority of women endorsed PP-PTSD following successful birth. Acute PP-PTSD rates were between 4.6 and 6.3%, and endorsement of clinically significant PP-PTSD symptoms was identified in up to 16.8% of women in community samples of high quality studies. Negative subjective experience of childbirth emerged as the most important predictor. Endorsement of PTSD before childbirth contributed to PP-PTSD; nevertheless, women without PTSD also exhibited PP-PTSD, with acute rates at 4.6%, signifying a new PTSD onset in the postpartum period.

**Conclusion:** Although the majority of women cope well, childbirth for some can be perceived as a highly stressful experience and even result in the development of PP-PTSD symptoms. More research is needed to understand postpartum adaption and childbirth-related posttraumatic stress outcomes.

## Introduction

Many people think of childbirth as a uniformly happy event. This belief may be responsible for the paucity of research into its possible deleterious outcomes. As unpleasant as it may be to face this, such research as exists indicates that childbirth may have negative (as well as positive) psychological effects. Postpartum women may experience psychological distress, and some may even develop mental disorders. Although postpartum depression has been extensively described (O'Hara and McCabe, 2013), accumulating data suggests that new mothers may also exhibit a posttraumatic stress response induced by the childbirth experience and may even suffer from childbirth-related postpartum traumatic stress disorder (PP-PTSD) following successful birth (Olde et al., 2006; Grekin and O'Hara, 2014).

As many as a third of women rate their delivery as psychologically traumatic (Ayers and Pickering, 2001), and as many as one quarter report some component of clinically significant PP-PTSD symptoms (Czarnocka and Slade, 2000). Co-morbidity of PP-PTSD and postpartum depression is high, as evident in up to 72% of cases of women endorsing PP-PTSD (Yildiz et al., 2017), and some may also endorse suicide ideation, as evident in 23% of cases (Dekel et al., in preparation). PP-PTSD may impair maternal bonding and indirectly have adverse affects on infant health (Williams et al., 2016). Hence, PP-PTSD is a problem deserving of attention.

A stress-diathesis model has been proposed to understand the factors giving rising to PP-PTSD (Ayers, 2004). Accordingly, PP-PTSD is the outcome of the interplay between pre-birth vulnerability factors, risk factors in birth, and factors after birth (Ayers et al., 2016). Factors such as pre-existing maternal psychiatric problems, a negative birth experience, and lack of social support, have been consistently reported as commonly endorsed risk factors (e.g., Andersen et al., 2012). These risks are broadly comparable with factors associated with PTSD in relation to other stressors (Ozer et al., 2003).

Much less agreement concerns the estimates of the incidence of PP-PTSD. PP-PTSD rates are between 1 and 30%, too wide a range to be very useful (Andersen et al., 2012; Grekin and O'Hara, 2014). Partial PP-PTSD, i.e., PTSD symptoms occurring at a clinically significant level, has also been noted, but again with a similar pattern of widely estimated rates (Czarnocka and Slade, 2000; Soet et al., 2003; Polachek et al., 2012).

Several review studies have been conducted on the topic. Olde et al. (2006) groundbreaking qualitative review of 19 studies published between 1997 and 2003 identified PP-PTSD following successful birth in up to 5.6% of women at 6 weeks postpartum. A subsequent review by Andersen et al. (2012) of 31 studies between 2003 and 2010 utilized a study quality ranking. They noted PP-PTSD rates in high quality studies were up to 4.6% between three and 12 months postpartum. Recent reviews importantly targeted community in comparison to at-risks samples (Grekin and O'Hara, 2014). As might be expected, PP-PTSD rates were approximately fivefold higher (15.7%) in at-risk groups than in the community (3.1%). Yildiz et al. (2017) report similar results from reviewing 28 studies using a diagnostic measure of PTSD, documenting once again a wide PP-PTSD rate variability among the studies.

These reviews support the existence of PTSD following childbirth; however, some clarification remains. While earlier reviews reported on the prevalence of PTSD during the postpartum period, it is not clear whether the studies were limited to those reporting on PTSD induced by childbirth. To our knowledge, a review comparing PP-PTSD prevalence in the context of prior PTSD versus a first time onset in the postpartum period was not conducted to-date. Such analysis is important to fully grasp the magnitude of the childbirth stressor.

PP-PTSD can be conceptualized in at least two ways. PTSD related to the childbirth event may develop in the context of endorsement of existing posttraumatic stress syndrome before childbirth. An estimated lifetime prevalence of PTSD in women is 10.4% and those who develop symptoms are highly susceptible to PTSD endorsement in relation to subsequent traumatic events (Kessler et al., 1995). PTSD is associated with a negative appraisal of subsequent events (Ehlers and Clark, 2000). In accord with Ayers (2004) the vulnerability factor of prior PTSD is likely to interact with birth events to determine appraisal of birth as traumatic and development of a traumatic stress responses. Thus, prepartum PTSD symptoms may continue into the postpartum period and trigger PP-PTSD. In contrast, PP-PTSD may signify the occurrence of first-time posttraumatic stress syndrome onset. Having a difficult childbirth experience (subjective or objective) for some women might be extreme enough to result in PP-PTSD without having prior PTSD symptoms. The childbirth event would then be regarded as the index event of traumatic exposure, although other vulnerability factors are possible. Women with a history of trauma exposure, who do not exhibit prepartum PTSD, may be a special case. Trauma history may reduce the ability to cope with subsequent stressful events (Dekel et al., 2013a), as noted in a sensitization effect (Breslau et al., 1999; Dekel et al., 2013b). Thus, women with trauma history may exhibit a delayed PTSD response induced by childbirth.

Another factor to consider in understanding incidences of PP-PTSD is the passage of time since childbirth. Although the majority of individuals endorse posttraumatic stress symptoms (i.e., PTS) in the immediate aftermath of trauma, symptoms subside for most survivors and a significant minority fails to recover (Bonanno et al., 2005; Shalev, 2009). Previous studies assessed rates of PP-PTSD at different time points ranging from the first days following childbirth to several months, which may account for the wide prevalence rates among the studies.

Thus, in the following systematic review, we examined the prevalence rates of PP-PTSD with a focus on the time period of symptoms endorsement following childbirth. We set to examine PP-PTSD in relation to the endorsement of new posttraumatic stress syndrome as well as in relation to prior PTSD. We also set to examine the risk factors involved in the development of PP-PTSD.

## Methods

### Study criteria selection

The search of literature targeted a wide range of studies, published from January 1980 until 1 August 2016. A search of PubMed and PsychInfo databases was performed using any combination of keywords: posttraumatic stress, posttraumatic stress disorder, traumatic stress with childbirth, PTSD, postpartum, or postnatal. Studies were also found through the “Related Citations” function on PubMed and through the reference lists of previously identified studies. Previous reviews on PTSD following childbirth were another basis to identify studies.

Studies included in our review were quantitative studies, published in English, and met the following criteria: (a) full-term successful births; (b) indication of prevalence of PP-PTSD, at a maximum initial assessment of 6 months postpartum; and (c) PP-PTSD stressor specified in relation to childbirth.

A total of 93 articles were identified via the search criteria. Of this total, 57 articles were removed either for failure to meet criteria or to avoid duplicate samples. The final review included 36 articles, with 32 representing community samples and four representing at-risk groups.

### Study rating system

A quality rating system modeled off Andersen et al. (2012) was utilized to assess identified studies. This system is based in part upon the Meta-analysis of Observational Studies in Epidemiology (MOOSE) technique (Stroup et al., 2000). It is designed to assess study quality by accounting for methodological differences among the studies. In this review, several factors were assessed to determine study quality such as sample size, method of data collection, measurement used, etc. Table 1 indicates the full list of factors, their corresponding items, and the point value assigned to each item. For example, the factor Data Collection included four items: single-item measures, study-specific measures, self-report questionnaire, and clinical interview. Single-item measures were assigned a point value of one, leading up to clinical interview, which was assigned a point value of four. Thus, a study would be given a score ranging from one to four for data collection. The sum of each factor resulted in an overall study quality score, ranging from 2 to 27 points.

**Table 1:** **Quality Coding System Criteria**.

**I. DATA COLLECTION**	
Single item measures	0
Study-specific measures	1
Self-report questionnaire	2
Clinical interview	3
**II. SAMPLE SIZE**	
≤29	0
30–99	1
100–199	2
200–399	3
≥400	4
**III. NATURE OF SAMPLE**	
Self-selective	0
Target/risk group	2
Representative	4
**IV. INITIAL RESPONSE RATE**	
Cross-sectional only	0
< 50%	1
50-75%	2
> 75%	3
**V. PRESENCE OF PRE-PARTUM MEASUREMENTS**	
No	0
Yes	1
**VI. HISTORY OF TRAUMA**	
No	0
Yes	1
**VI. HISTORY OF PTSD**	
No	0
Yes	1
**VII. POST-PARTUM COLLECTION POINTS**	
One Time	1
Two Times	2
Three+ Times	3
**VIII. PTSD ASSESSMENT**	
Post-Partum only	0
Pre- and Post-Partum	1
Post-Partum and IOC	2
Pre-, Post-, and IOC	3
**IX. INITIAL POST-PARTUM ASSESSMENT**	
Years	1
Months	2
Weeks	3
Days	4
**SUM OF POINTS**	2–27

*The drop-out rate among studies was evaluated after the initial time point. The prevalence of high drop-out rates in some studies could indicate selection bias. A sum of points was calculated based on the coding factors below. The minimum and maximum scores achievable were 2 and 27, respectively. Studies scoring 2–18 points were ranked as B, and 19–27 points were ranked as A. IOC, Index of Childbirth*.

According to their study quality score, each study was ranked as either A or B. The A category included studies with the highest methodological ranking, and, for the relevance of this review, those that controlled for PTSD symptom endorsement before childbirth (“controlled studies”). The B category included studies that may have received a lower quality score due to smaller sample size, fewer postpartum time assessments, lack of perinatal assessments, etc., or due to less relevance to this review. All studies in B did not control for PTSD before childbirth (“uncontrolled studies”).

Two research assistants analyzed and rated the 36 identified articles. Inter-rater agreement between the research assistants was high and reached 92%. If disagreements in coding arose, the disagreement was discussed and resolved to reach a consensus.

In the next step, we calculated PTSD prevalence rates within studies in Groups A and B by taking into consideration the time of postpartum assessment. In accordance with PTSD DSM-4 (American Psychiatric Association, 2000), PP-PTSD assessed more than 1 month and up to 3 months postpartum was labeled “acute PP-PTSD,” and assessment more than 3 months and up to 6 months postpartum was labeled “chronic PP-PTSD.” In addition, PP-PTSD symptoms measured within 1 month postpartum were labeled postpartum posttraumatic stress, i.e., PP-PTS. PTSD rate for each time point was created by computing the mean PTSD value. We also computed the overall PP-PTSD rate assessed by averaging the time categories (PP-PTS + acute PTSD + chronic PTSD).

### Risk factor analysis

Risk factors for PP-PTSD, if presented by the studies, were identified and analyzed. To be included in the analysis, risk factors had to be of statistical significance. Each identified risk factor was assigned a point value based on the group rank of the study. For example, a risk factor identified from a Group A study was assigned a point value of two. Risk factors identified from Group B were assigned a point value of one. If multiple studies reported the same risk factor, that risk factor received a summed score.

Risk factors of similar nature were then grouped into categories. For example, risk factors such as prenatal depression and perinatal anxiety were grouped under the category “Maternal Mental Health.” Five categories were created. The category score was the sum of the individual risk factors, and was above 15 for each category. A miscellaneous category was added to account for risk factors that did not fall within the five identified categories.

## Results

### Study classification

The identified 36 articles meeting inclusion criteria were categorized into groups A and B in accordance with the proposed coding system. Group A included 23 studies which were categorized according to whether they controlled for endorsement of PTSD prior to childbirth (controlled) or not (uncontrolled) (See Table 2). Group B included 13 studies (See Table 3).

**Table 2:** **Group A Studies**.

**Study**	**Sample size**	**Type of sample**	**Study site**	**Measures of PP-PTSD**	**Measurement times postpartum**
Abedian et al., 2013^b^	*n* = 100	Women with preeclampsia	Iran	PPQ	6 weeks
Alcron et al., 2010^a^^,^^b^	*n* = 866	Community	Australia	PDS	T1: 4-6 weeks T2: 12 weeks T3: 24 weeks
Ayers et al., 2009^a^	*n* = 1423	Community	UK	PDS	3-12 months
Ayers and Pickering, 2001^b^	*n* = 289	Community	UK	MMPI-2 PTSD Scale	T1: 6 weeks T2: 6 months
Boorman et al., 2014^a^	*n* = 890	Community	Australia	Criterion A1 and A2 assessment	14 days
Cigoli et al., 2006^a^	*n* = 160	Community	Italy	PTSD-Q	3–6 months
Cohen et al., 2004^a^	*n* = 200	Community	Canada	DTS	8–10 weeks
Czarnocka and Slade, 2000^a^	*n* = 264	Community	UK	PTSD-Q	6 weeks
Davies et al., 2008^b^	n = 211	Community	UK	PTSD-Q	6 weeks
Ford et al., 2010^b^	*n* = 138	Community	UK	PDS	T1: 3 weeks T2: 3 months
Garthus-Niegel et al., 2014^a^	*n* = 1700	Community	Norway	IES	8 weeks
Ionio and Blasio, 2014^a^	*n* = 19	Community	Italy	PPQ	T1: 2 days T2: 2 months
Milosavljevic et al., 2016^b^	*n* = 1 26	community	Serbia	CAPS	T1: 1 month T2: 2 months T3: 3 months
Polachek et al., 2012^a^	*n* = 89	Community	Israel	PDS	1 month
Schwab et al., 2012^a^^,^^b^	*n* = 56	Community	Iran	PDS	6 weeks
Seng et al., 2013^a^^,^^b^	*n* = 566	Community	USA	NWS PTSD module	6 weeks
Soderquist et al., 2006^a^	*n* = 1224	Community	Sweden	TES	T1: 1 month T2: 4 months T3: 7 months T4: 11 months
Son et al., 2005^a^	*n* = 248	Community	Netherlands	IES	T1: 3 months T2: 6 months T3: 12 months
Sumner et al., 2012^b^	*n* = 206	Low-income Latinas	South America	PCL-C	T1: 7 months T2: 13 months
Verreault et al., 2012^a^	*n* = 308	Community	Canada	SCID-I; MPSS-SR	1 month
Vossbeck-Elsebusch et al., 2014^a^^,^^b^	*n* = 224	Community	Germany	PDS	1–6 months
White et al., 2006^a^	*n* = 318	Community	Australia	PSS-SR	T1: 6 weeks T2: 6 months T3: 12 months
Zambaldi et al., 2011^a^^,^^b^	*n* = 400	Community	Brazil	M.I.N.I.	2–26 weeks

*PP-PTSD endorsement was defined as reaching a score above the cut-off for PTSD symptom severity, or as being in accord with DSM PTSD symptom criteria (DSM-4 or DSM-5). CAPS, Clinician-Administered PTSD Scale; DTS, Davidson Trauma Scale; IES, Impact of Event Scale; M.I.N.I., Mini-International Neuropsychiatric Interview; MMPI-2 PTSD Scale, Minnesota Multiphasic Personality Inventory-2 Posttraumatic Stress Disorder Scale; MPSS-SR, Modified PTSD Symptom Scale Self-Report; NWS PTSD Module, National Women's Study PTSD Module; PCL-C, PTSD Checklist-Civilian Version; PDS, Posttraumatic Stress Diagnostic Scale – Self-report version; PPQ, Perinatal PTSD Questionnaire; PSS-SR, Posttraumatic Stress Symptom Scale – Self-report version; PTSD-Q, Posttraumatic Stress Disorder Questionnaire; SCID-I, Structured Clinical Interview for DSM-4; TES, Traumatic Event Scale*.

a *Studies that did not control for prior PP-PTSD*.

b *Studies that controlled for prior PP-PTSD*.

**Table 3:** **Group B Studies**.

**Study**	**Sample size**	**Type of sample**	**Study site**	**Measures of PTSD**	**Measurement times post-partum**
Adewuya et al., 2006	*n* = 876	Community	Nigeria	M.I.N.I	6 weeks
Beck et al., 2011	*n* = 903^a^	Community	USA	PSS-SR	6 months
Ghorbani et al., 2014	*n* = 82^b^	Community	Iran	PSS	<2 months
Leeds and Hargreaves, 2008	*n* = 102	Community	UK	PCL; PPQ	6–12 months
Modarres et al., 2012	*n* = 400	Women with traumatic delivery	Iran	PSS-I	6–8 weeks
Olde et al., 2005	*n* = 140	Community	Netherlands	PSS-SR	T1: 1–7 days T2: 3 months
Parfitt and Ayers, 2009	*n* = 152	Community	UK	PDS	10 months^c^
Ryding et al., 1997	*n* = 25	Community	Sweden	CI; FET	T1: 1–9 days T2: 1–2 months
Sexton et al., 2015	*n* = 214	Women who had experienced child abuse and neglect^d^	USA	NWS PTSD	4 months
Shaban et al., 2013	*n* = 600	Community	Iran	PSDS	6–8 weeks
Soet et al., 2003	*n* = 103	Community	USA	Telephone interview including TES	1 months
Wijma et al., 1997	*n* = 1640	Community	Sweden	TES	T1: 1–13 months
Zaers et al., 2008	*n* = 60	Community	Germany	PDS	T1: 6 weeks T2: 6 months

*PP-PTSD endorsement was defined as reaching a score above the cut-off for PTSD symptom severity, or as being in accord with DSM PTSD symptom criteria [DSM-4 (American Psychiatric Association, 2000) and DSM-5 (American Psychiatric Association, 2013)]. CI, Clinical Interview; FET, Fisher's Exact Test; M.I.N.I., Mini-International Neuropsychiatric Interview; NWS PTSD, National Women's Study PTSD Module; PCL, Posttraumatic Stress Disorder Checklist; PDS, Posttraumatic Stress Diagnostic Scale; PPQ, Perinatal PTSD Questionnaire; PSDS, Posttraumatic Stress Disorder Symptom Scale; PSS, Posttraumatic Stress Disorder Symptoms Scale; PSS-I, Posttraumatic Symptom Scale-Interview; PSS-SR, Posttraumatic Stress Symptom Scale-Self-report version; TES, Traumatic Event Scale*.

a *Sample that completed PTSD assessment*.

b *Sample that only includes full-term births*.

c *Mean postpartum measurement time*.

d *Two-thirds of women in the sample had experienced child abuse and neglect*.

### PP-PTSD prevalence

All 36 articles included in this review reported the prevalence of childbirth-related PP-PTSD, with 18 studies also reporting the prevalence of partial PP-PTSD.

Table 4 presents PP-PTSD prevalence according to group classification (A and B), time of postpartum assessment, and type of sample (community vs. at-risk). As indicated previously: “PP-PTS” noted was for symptoms ≤1 month postpartum; “acute PP-PTSD” for symptoms >1month postpartum but <3 months postpartum; and “chronic PP-PTSD” for symptoms >3 months and ≤6 months. Overall, PP-PTSD and partial PTSD referred to symptoms across time points.

**Table 4:** **Prevalence rates for PP-PTSD in relation to PTSD history and time of assessment**.

	**A**		**B**
**PP-PTSD**	**Controlled**	**Uncontrolled**	**Uncontrolled**
PP-PTS	0.80%	6.70% (1.3–14.3)	1.00% (0–1.9)
Acute PP-PTSD	10.10%^a^; 4.60%^*b*^ (1.2–37.8)^a^; (1.2–11.2)^b^	6.30% (0–21.5)	11.10%^a^; 8.10%^b^ (1.2–21.5)^*a*^; (1.2–17.2)^b^
Chronic PP-PTSD	3.90%^a^; 1.80%^b^ (0–9.65)^a^; (0–3.1)^b^	3.40% (0.9–6)	6.70%^a^; 4.60%^b^ (1.2–19.6)^*a*^; (1.2–6.4)^b^
Overall	4.90%^a^; 2.40%^*b*^	5.50%	6.20%^a^; 4.50%^b^
Partial PP-PTSD	9.60% (1.3–21.3)	16.80% (6.1–28.8)	27.30% (9.1–19.6)

*Numbers reflect percentage of women who endorse PP-PTSD symptoms. Numbers in parentheses indicate range of prevalence rates reported. PP-PTSD, Postpartum Posttraumatic Stress Disorder. PP-PTS, PTSD symptoms assessed within one month postpartum. Acute PP-PTSD, PTSD assessed more than one month postpartum but less than three. Chronic PP-PTSD, PTSD assessed from three months postpartum on. Controlled and uncontrolled studies, studies that did and did not control for PTSD prior to childbirth*.

a Population sample including both community and at-risk groups;

b *Community samples*.

For “Controlled” studies in Group A, the overall PP-PTSD prevalence was 4.9%, including at-risk samples. Though PP-PTS rates were 0.8%, acute PP-PTSD rates were much higher, with 4.6% in community samples and 10.1% including at-risk. Chronic PP-PTSD rates were 1.8% in the community sample and 3.9% including at-risk samples, suggesting a decline in PP-PTSD endorsement. Overall partial PP-PTSD was 9.6%. For example, a controlled community study (*N* = 211 of primi- and multiparas women who underwent various modes of delivery with healthy outcomes) reported rates of full and partial PP-PTSD at 3.8 and 21.3%, respectively, at 6 weeks postpartum (Davies et al., 2008).

For “Uncontrolled” studies in Group A, the overall PP-PTSD prevalence was 5.5%. Rates of PP-PTS and acute PP-PTSD were similar, at 6.7 and 6.3%, respectively. Again, chronic PP-PTSD rates were lower at 3.4%. The overall partial PP-PTSD rate, as might be expected, was much higher, at 16.8%; three times higher than the rates for overall PP-PTSD. For example, a uncontrolled study (*N* = 308 of community sample) reported partial and full PP-PTSD rates of 7.6 and 16.6%, respectively, at 1 month (Verreault et al., 2012).

In Group B, the overall PP-PTSD prevalence including at-risk samples was 6.2%. The prevalence rates also varied according to time of assessment, with lowest rates found for PP-PTS and highest for acute PP-PTSD. PP-PTS prevalence was 1.0%, whereas acute PP-PTSD was 8.1% in the community sample and 11.1% when including at-risk groups. Chronic PP-PTSD prevalence was lower than acute, with rates of 4.6% in community samples and 6.7% including at-risk samples. Rates of partial PP-PTSD were highest at 27.3%.

### PP-PTSD risk factors

Five categories of risk factors were identified: negative perception of childbirth, maternal mental health, trauma history, and PTSD, delivery mode and complications, and low social support. Table 5 presents the categories and their risk factors.

**Table 5:** **Risk factors associated with PP-PTSD**.

**Subjective experience of childbirth**	**28**	**Maternal mental health**	**25**	**Trauma history and PTSD**	**24**	**Delivery mode and complications**	**22**	**Low social support**	**17**
Negative delivery experience	8	Prenatal depression	8	Previous traumatic events	9	Emergency caesarean section	7	Staff	6
Fear of childbirth for self and/or baby	6	History of psychological problems	7	Childhood sexual trauma	5	Complications with pregnancy and/or baby	5	Overall	5
Low internal locus of control during childbirth	5	Perinatal anxiety	5	Prenatal PTSD	2	Instrumental delivery	4	Partner	4
Post-traumatic cognitions	4	Perinatal somatoform	3	Previous traumatic birth experience	2	Planned caesarean birth	2	Family	2
Pain in labor	3	Postpartum depression	1	Pre-traumatic stress in pregnancy	2	Long labor duration	1		
Low coping ability	1					Manual removal of placenta	1		
Perinatal dissociation	1					Preterm birth	1		
Unexpectedness of procedures	1					Pressure to have an induction and epidural analgesia	1		

*PP-PTSD, Postpartum Posttraumatic Stress Disorder*.

Factors relating to negative subjective experience of childbirth received the highest weighted score of 28 points, and were thus identified as the most potent predictors of PP-PTSD. The most significant risk factor, reported by five studies, was negative delivery experience (Wijma et al., 1997; Son et al., 2005; Zaers et al., 2008; Verreault et al., 2012; Garthus-Niegel et al., 2014). Fear of childbirth for the self and/or baby and low internal locus of control during childbirth were also significant factors noted in this category, and were reported by several studies (Czarnocka and Slade, 2000; Soet et al., 2003; Adewuya et al., 2006; Soderquist et al., 2006; Leeds and Hargreaves, 2008; Garthus-Niegel et al., 2014).

The next most significant group of risk factors included those pertaining to maternal mental health. This group received a weighted score of 25 points. The presence of prenatal depression was the most commonly noted factor within this group, as documented in five studies (Cohen et al., 2004; Soderquist et al., 2006; Leeds and Hargreaves, 2008; Sumner et al., 2012; Shaban et al., 2013). Having a history of psychological problems prior to pregnancy was found to significantly contribute to the endorsement of PP-PTSD as well (Soderquist et al., 2006; Leeds and Hargreaves, 2008; Zaers et al., 2008; Zambaldi et al., 2011; Boorman et al., 2014). Perinatal somatoform and anxiety were also significant factors cited by six studies (Soet et al., 2003; Olde et al., 2005; Zaers et al., 2008; Zambaldi et al., 2011; Verreault et al., 2012; Shaban et al., 2013). Finally, acute postpartum depression symptoms were found to predict later PP-PTSD (Beck et al., 2011).

Factors falling within the trauma history and PTSD category comprised the third highest weighted score of 24 points. A history of trauma exposure of any type was the most commonly reported risk factor as noted in three studies (White et al., 2006; Zaers et al., 2008; Ayers et al., 2009). Childhood sexual trauma in particular was found to be a highly cited factor, as well as pre-traumatic stress in pregnancy (Soderquist et al., 2006; Lev-Wiesel and Daphna-Tekoah, 2010; Verreault et al., 2012; Sexton et al., 2015). As might be expected, the endorsement of PTSD during pregnancy was a strong predictor of PP-PTSD (Seng et al., 2013). Trauma related to interpersonal violence and having a previous traumatic birth experience were other frequently reported factors (Polachek et al., 2012; Sumner et al., 2012).

Risk factors related to delivery mode and complications constituted the fourth highest weighted category of 22 points. Emergency cesarean section and complications with the pregnancy and/or baby were the most cited risk factors in this category, reported by seven studies total (Cohen et al., 2004; Adewuya et al., 2006; Zambaldi et al., 2011; Modarres et al., 2012; Boorman et al., 2014; Vossbeck-Elsebusch et al., 2014; Milosavljevic et al., 2016). Two studies also noted instrumental deliveries as a significant risk factor (Adewuya et al., 2006; Milosavljevic et al., 2016).

The final category pertained to low levels of social support, and received a weighted score of 17 points. Three studies referred to low levels of social support in general as a risk factor (Soet et al., 2003; Ford et al., 2010; Sumner et al., 2012). The remaining studies were more specific in regards to the support group, with one study reporting low family support, three studies reporting low staff support, and three studies reporting low partner support (Czarnocka and Slade, 2000; Cigoli et al., 2006; Parfitt and Ayers, 2009; Beck et al., 2011; Ford and Ayers, 2011). Previous counseling related to childbirth or pregnancy was also a contributing factor (Soderquist et al., 2006).

Finally, we identified common risk factors that did not fit into the noted risk factors categories. These factors largely pertain to demographics and included the risk factors such as young age, low income, primiparity, and multiparity (Zambaldi et al., 2011; Abedian et al., 2013; Boorman et al., 2014; Vossbeck-Elsebusch et al., 2014).

## Discussion

Posttraumatic stress syndrome related with the childbirth experience has been largely overlooked. That a mostly voluntary event implicated in reproduction can trigger PP-PTSD does not reconcile with the positive notion of childbirth. Yet, childbirth involves drastic physiological changes of hormonal imbalance, blood and body weight loss, increased cardiac output, and is often accompanied by acute bodily pain and sleep deprivation. Not surprisingly, a full-term delivery with healthy outcomes may nonetheless be associated with threat on bodily integrity and a sense of fear and loss of control.

There are two competing theories on childbirth-related PTSD. First, PP-PTSD signifies amplification of pre-existing traumatic stress symptoms. Second, PP-PTSD indicates a new PTSD onset induced by the childbirth experience rather than continuation of perinatal PTSD. We explored predictions from these theories by analyzing 36 articles reporting the rates of PP-PTSD symptoms associated with the experience of healthy, full-term birth.

Overall, the prevalence rates of PP-PTSD, in its acute form (between 1 and 3 months postpartum) were 5–8% in community samples. The prevalence of clinically significant symptoms of PP-PTSD, was higher, and ranged between 9.6 and 27.3%. It is estimated that four million babies are born in the States each year. While our results suggest that at a minimum only 5 out of 100 women will experience a sort of PP-PTSD, when translated to the larger sample of four million, this is roughly two hundred thousand mothers at-risk.

New PTSD onset following childbirth was noted in the studies reviewed here, suggesting that for some women childbirth can be perceived as a highly stressful experience capable of triggering PTSD without having prior PTSD. In fact, rates of new PTSD onset evident in samples of women without PTSD before childbirth were slightly lower than in samples including women with or without PTSD history, 4.6 vs. 6.3% for acute PP-PTSD in studies of high quality. Although these findings may be attributed to differences in the nature of the samples, we applied a quality approach to compare between the studies. Alternatively, the findings may suggest that for a significant sub-group of women, PTSD symptoms do not carry on from pregnancy to childbirth but rather develop for the first time in the immediate postpartum period. Although several reviews have been conducted on PTSD in relation to childbirth, this is the first attempt to distinguish between syndromes of new and prior PTSD endorsement.

Various risk factors were also reviewed and identified as potentially predictive of the development of PP-PTSD, mainly pre-trauma related factors and peritraumatic aspects related to the childbirth. Although predisposing mental health conditions and prior trauma were associated with subsequent PP-PTSD, peritraumatic factors and early emotional symptoms emerged as the strongest predictor. Obstetrical factors and related complications, which may entail threat to life of mother and/or baby, were ranked as less important. These findings accord with the previous reviews (Andersen et al., 2012) and are also in line with the literature of PTSD in relation to other stressors (Ozer et al., 2003). It has been documented that the subjective experience of the traumatic event is a more important factor in predicting PTSD than its objective stressor severity (Bowman, 1999; Dekel et al., 2016a). Subjective negative childbirth experience, as our data reveals, pertains to having a negative appraisal of the event (primary appraisal) and of one's ability to cope with the stressor (secondary appraisal) (Lazarus, 1981). These kinds of negative appraisals have been linked with the development and endurance of PTSD following traumatic experiences (Dunmore et al., 2001).

Several limitations in this review should be noted. Although identified studies used well-validated measures of PP-PTSD symptoms, whether participants had clinically diagnosed PP-PTSD was assessed by some studies but not in all. While we identified common risk factors for PP-PTSD, the interplay between the factors was not assessed, and there might be other unknown factors that have not been studied yet. Analysis of quantitative studies allowed for study comparison; however, qualitative studies may have offered important information as well. We applied a quality rating to compare and integrate studies, but might have overlooked factors that may bias the results. Although we mainly reviewed community samples, important confounding factors such as age, medical issues during birth, mode of delivery, and peripartum anxiety, were not taken into account when reporting PP-PTSD rates. We included samples derived from different world regions, which offers a broad perspective on PP-PTSD but might create wide variations in prevalence rates. Ideally, we would have targeted large, prospective, longitudinal studies to clarify childbirth-related PTSD with/without PTSD or trauma history and the moderating factor of culture (Dekel et al., 2016b). Future studies are warranted to examine the various pathways for the development of PP-PTSD and its symptom trajectory.

Within the context of these limitations, the current review provides important evidence for the endorsement of PTSD related with childbirth. Although there has been controversy regarding whether PTSD could be induced by childbirth, our findings indicate that a significant sub-group of women with and without prior PTSD may develop PP-PTSD of an enduring nature. The development of PTSD in relation to a relatively predicted event (i.e., birth) during routine clinical care offers the opportunity to potentially identify women at high risk for PP-PTSD and provide specific preventive interventions, both of which are currently lacking. As this study demonstrates, having a negative childbirth experience is an important factor implicated in PP-PTSD. Childbirth accounts may be a useful tool to identify at-risk women in the immediate peripartum period and to intervene accordingly. At a broader level, our findings suggest that by nature women are resilient and cope well with the childbirth experience. Childbirth is not inevitably appraised as traumatic. Examining biological factors underlining positive adaptation and posttraumatic stress reactions is likely to provide insight into this under-researched scientific territory.

## Author contributions

SD supervised coding of articles and data analysis and headed manuscript writing. CS identified and coded articles, completed data analysis, and contributed to manuscript writing. GD identified and coded articles.

## Funding

This work was supported by MGH ECOR Claflin Distinguished Scholar Award and the Brain and Behavior Research Foundation (NARSAD) Grant awarded to SD.

### Conflict of interest statement

The authors declare that the research was conducted in the absence of any commercial or financial relationships that could be construed as a potential conflict of interest.
